# Establishment and characterization of a novel hilar cholangiocarcinoma cell line, CBC3T-1

**DOI:** 10.1007/s13577-023-01003-4

**Published:** 2023-11-15

**Authors:** Mingzhen Bai, Ningzu Jiang, Wenkang Fu, Chongfei Huang, Liang Tian, Ningning Mi, Long Gao, Haidong Ma, Yawen Lu, Jie Cao, Chao Zhang, Ping Yue, Yong Zhang, Yanyan Lin, Wenbo Meng, Xun Li

**Affiliations:** 1 The First Clinical Medical College of Lanzhou University, Lanzhou, 730030 Gansu China; 2 Department of General Surgery, The First Hospital of Lanzhou University, Lanzhou, 730030 Gansu China; 3 Gansu Province Key Laboratory of Biological Therapy and Regenerative Medicine Transformation, Lanzhou, 730030 Gansu China

**Keywords:** Biliary, Hilar cholangiocarcinoma, Cell line, Xenograft, Somatic mutation

## Abstract

Cholangiocarcinoma (CCA) is a group of malignant heterogeneous cancer arising from the biliary tree. The tumor is characterized by insidious onset, high degree of malignancy, poor prognosis, and high recurrence rate. Immortalized cancer cell lines are the best and easiest models for in vitro cancer research. Here, we established a naturally immortalized highly tumorigenic hilar cholangiocarcinoma (hCCA) cell line, CBC3T-1. The CBC3T-1 cell line was cultured for over 60 passages. Thorough analysis showed that CBC3T-1 cells share characteristics similar to original tumor cells from patients with cholangiocarcinoma and display a stable phenotype, including features of epithelial origin, stem cell-like properties, as well as a high invasive and migratory capability and tumorigenicity in mice. Furthermore, this cell line showed the best sensitivity to paclitaxel, followed by gemcitabine. RNA sequencing and whole‑exome sequencing showed that cancer-associated pathways and somatic mutations played a dominant role in the development of CCA. We established and characterized a new hCCA cell line, CBC3T-1, which contributes to a better understanding of bile duct cancer, and can be used to study tumorigenesis and progression and the role of anticancer drugs.

**Supplementary Information:**

The online version contains supplementary material available at 10.1007/s13577-023-01003-4.

## Introduction

Cholangiocarcinoma (CCA) is a lethal hepatobiliary malignancy with limited therapeutic options [1, 2]. According to the anatomical location, CCA is classified as intrahepatic cholangiocarcinoma (iCCA) and extrahepatic cholangiocarcinoma (eCCA), which can be further divided into hilar cholangiocarcinoma (hCCA) and distal cholangiocarcinoma (dCCA) [3, 4]. The incidence of CCA varies by geography and population, and the overall incidence continues to increase worldwide [5]. Globally, the incidence of CCA ranges from 0.3 to 6/100,000 inhabitants per year, with a mortality rate of 1–6/100,000 inhabitants per year [6]. In particular, Korea, Thailand, and China show a particularly high incidence of more than 6 cases/100,000 people per year [7]. The 5-year survival rate for CCA is less than 10% due to difficult diagnosis and limited treatment options [8, 9].

Patient-derived cancer models are valuable tools for elucidating the molecular mechanisms of cancer development and disease progression. Cell lines are effective in vitro model system that can be used to assess the characteristics of cancers [10]. There are approximately 112 CCA cell lines in the world, including 92 iCCA and 30 eCCA cell lines, which are distributed mainly in the United States, Japan, and Thailand [11]. Most of the cell lines used in studies have been maintained for decades, and they are susceptible to genotypic and phenotypic drift during successive passages [11, 12]. During embryogenesis, iCCA and eCCA subtypes differ in their tumorigenic processes [13]. However, our understanding of hCCA/dCCA pathogenesis remains limited due to the lack of eCCA research models.

Therefore, we established and biologically and molecularly characterized a new hCCA cell line, named CBC3T-1, from a Chinese patient. Our results suggest that the CBC3T-1 cell line is a useful model for studying hilar cholangiocarcinoma.

## Materials and methods

### Patient background

**Fig. 1:**
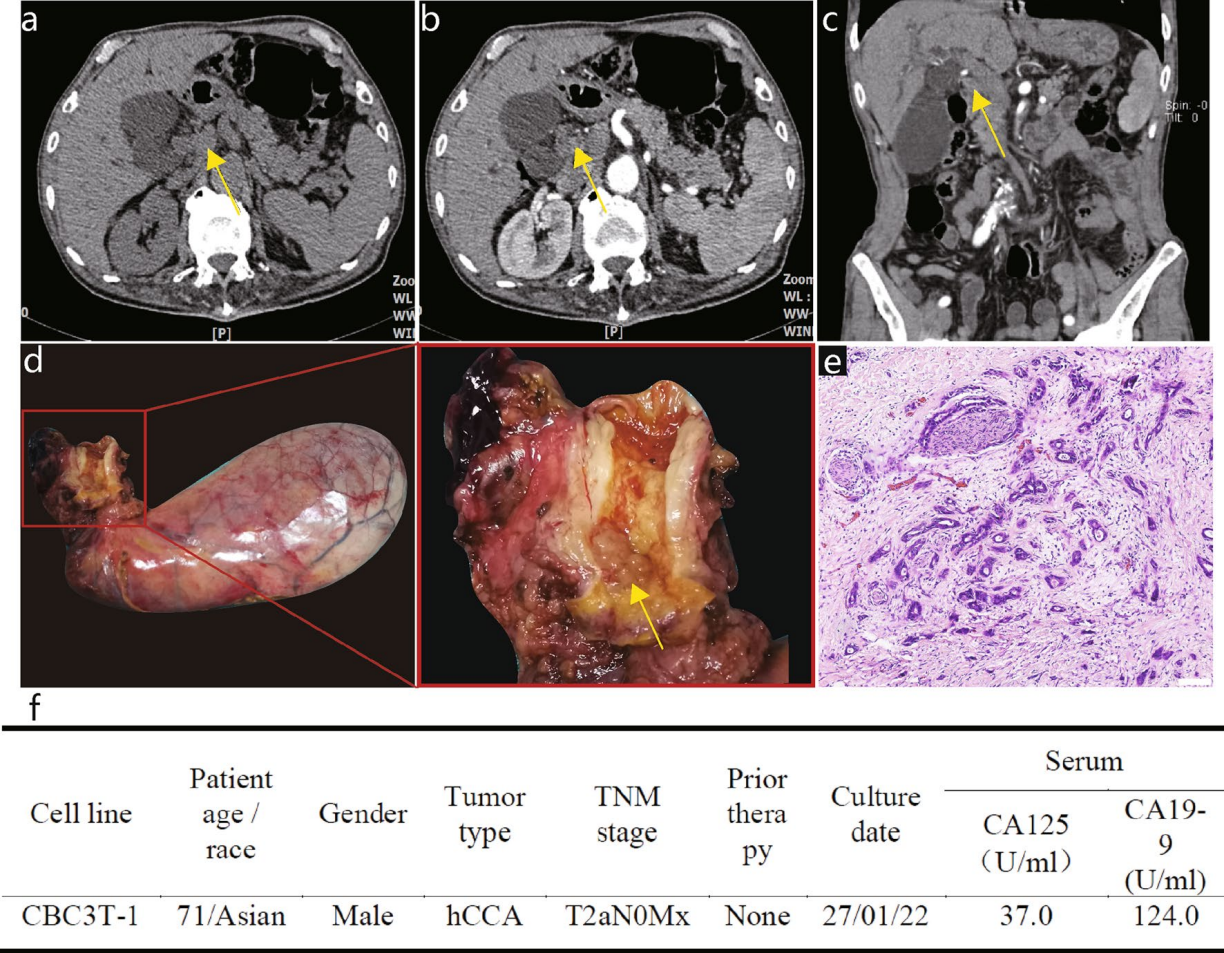
Clinical and pathological profile of CBC3T-1. Computed tomographic (CT) scan of the abdomen. Plain CT scan (**a**) showed circumferential thickening of the upper bile duct wall with luminal narrowing, while a contrast-enhanced CT scan (**b**, **c**) revealed that the tumor was slightly enhanced (yellow arrow). **d** General view of the surgically resected specimen. **e** H&E staining of primary tumor tissue. Scale bars 100 μm. **f** Clinicopathological profile of CCA patients

The donor patient was a 71-year-old male with hCCA. The patient had elevated tumor marker levels, and computed tomography (CT) images showed circumferential thickening and mild enhancement of the perihilar bile duct with luminal narrowing (Fig. 1a–c). A diagnosis of hCCA was considered. No preoperative radiotherapy or chemotherapy was administered, and the patient underwent resection of the hCCA. The tumor specimen showed a circumferential soft-tissue perihilar mass (Fig. 1d). The postoperative pathological diagnosis of this patient's tumor tissue was moderately differentiated adenocarcinoma of the bile duct (Fig. 1e).

### Cell culture

The human eCCA cell line TFK-1 and human biliary epithelial cell line HIBEpiC were purchased from the National Biomedical Experimental Cell Resource Bank of China (Beijing, China). The cells were cultured in RPMI 1640 medium (Gibco) containing 10% FBS (Gibco, USA), 100-U/mL penicillin, and 100-mg/mL streptomycin (Gibco, USA), and placed in an incubator with a constant temperature of 37 °C and 5% CO_2_.

### Primary cell culture

Immediately after surgery, specimens were obtained from the primary tumor tissue. The tissue was cut to a 1-mm^3^ size with scissors. Subsequently, the tissues were transferred to DMEM/F-12, (Gibco, USA) containing 200 U collagenase II and digested at 37 °C for 15–30 min, after which a single-cell suspension was obtained by filtering the supernatant through a 100-μm cell strainer. The filtrate was centrifuged (300 g/5 min), and the cell precipitate was resuspended in DMEM/F-12 supplemented with 10% FBS and 100-mg/ml Primocin™ (InvivoGen, CA). Cells were incubated at 37 °C in a humidified atmosphere with a 5% CO_2_ incubator. All subsequent experiments were performed after 30 generations.

### Verification of cell lines by ATCC

The CBC3T-1 cell line was authenticated by ATCC. Briefly, genomic DNA was extracted from the CBC3T-1 cell line using a QIAamp DNA Mini Kit (Qiagen, The Netherlands). The alleles of 21 loci in CBC3T-1 cells (D19S433, D5S818, D21S11, D18S51, D6S1043, AMEL, D3S1358, D13S317, D7S820, D16S539, CSF1PO, Penta D, D2S441, vWA, D8S1179, TPOX, Penta E, TH01, D12S391, and D2S1338) were amplified by PCR and analyzed using an Applied Biosystems 3730xl DNA Analyzer (Applied Biosystems, USA). When the short tandem repeat (STR) data of CBC3T-1 cells in the databases of ATCC, DSMZ, and CELLOSAURUS were compared, the profile did not exactly match with any of the current data.

### Cell Counting Kit (CCK)-8 cell growth assay

Cells were seeded into 96-well plates at a density of 6 × 10^3^ cells per well. After incubation for 0, 24, 48, 72, and 96 h, Cell Counting Kit 8 (CCK-8) reagent (APExBio, USA) was added to the cells, and the cells were incubated for 2 h. The absorbance at 450 nm was measured.

### Mycoplasma detection by PCR

For the detection of cellular mycoplasma, the medium of CBC3T-1 cells was collected and assayed according to the mycoplasma detection kit (Invitrogen, CA). DNA fragments were imaged under UV irradiation.

### Chromosome karyotype analysis

Cells were incubated for 2 h using 0.4 µg/ml colchicine. Then cells were collected and incubated with 0.075 M KCl for 30 min (37 °C) and fixed 3 times with methanol: acetic acid (3:1) at room temperature for 10 min. Slides were then prepared and stained with Giemsa. Representative images of chromosome sets were obtained for karyotype analysis.

### Live cell imaging

Cells were digested and cultured in 96-well plates until they attached to the surface. Live cell imaging was performed using a Cytation 1 imaging system (Biotek, USA) under a 4 × objective. Label-free live cell proliferation measurements were achieved by capturing two high-contrast brightfield images at each 2-h time point. Images were processed with Gen 5.

### Spheroid formation assay

Cells were inoculated at a density of 1000 cells per well in an ultralow-attachment 96-well plate (Corning, USA) containing 10% FBS in DMEM/F-12. The cells were observed with a microscope on days 3, 7, and 14 of incubation.

### Wound-healing assay

Cells were inoculated in 6-well plates. When 95% confluence was reached, the cell monolayers were scratched with a P-200 pipette tip, and the scratched monolayers were gently washed three times with phosphate buffer solution (PBS). Medium containing 10% FBS was then added for further incubation. Images were captured at 0, 12, and 24 h, and the distance between the two wound edges was quantitatively assessed by measuring the entire area of the scratches with ImageJ software.

### Transwell migration/invasion assays

For Transwell plate migration assays, a total of 8 × 10^4^ cells were seeded in the upper chamber of an 8 μm Transwell plate (BD Biosciences, USA) with 100 μl of serum-free medium. In the lower chamber, 500 μl of medium containing 15% FBS was added. After 24 and 48 h of incubation, the cells in the upper chamber were carefully removed. Cells adhering to the membrane were fixed in 4% paraformaldehyde for 15 min and stained with 0.1% crystalline violet (Beyotime, China) for 15 min. For invasion assays, 50 µl of Matrigel (Corning, USA) diluted 1:4 with DMEM/F-12 was precoated in the upper chamber and seeded with 8 × 10^4^ cells in 100 µl of DMEM/F-12. The rest of the procedure was similar to that of the Transwell migration assay.

### Colony-forming assay

Cells were seeded in 6-well plates at a density of 700 cells/well in different complete media and allowed to form colonies for 14 days. Colonies were fixed with 4% paraformaldehyde for 15 min and stained with 0.1% crystal violet for 15 min. The results were photographed and observed using an inverted phase contrast microscope as described above and analyzed using ImageJ software.

### Sensitivity to anti‑cancer drugs

For testing of sensitivity to first-line clinical agents (oxaliplatin, cisplatin, gemcitabine, 5-fluorouracil, and paclitaxel) for CCA, CBC3T-1 cells were seeded at a density of 1 × 10^4^ cells per well in 96-well plates. The cells were cultured overnight and then treated with anticancer drugs for 72 h. Cell viability was determined using a Cell Counting Kit 8 (APExBIO, USA).

### Tumorigenicity in NOD/SCID mice

To study the tumorigenicity of the CBC3T-1 cell line, 4–6-week-old female NOD/SCID (nonobese diabetic/severe combined immunodeficient) mice were purchased from Beijing Weitong Lihua Experimental Animal Technology Co. Ltd. A total of 2 × 10^6^ cells were resuspended in 0.2 ml of DMEM/F-12 and Matrigel (Corning, USA) mixture and injected subcutaneously into each NOD/SCID mice. The animals were kept in a laminar flow cabinet under specific pathogen-free conditions. The mice were continuously monitored for tumor growth. After 21 days, tumor tissue was collected, measured and weighed, fixed in 10% formalin, embedded in paraffin, stained with hematoxylin and eosin (H&E), and subjected to immunohistochemistry (IHC). The animal experiment was performed in accordance with the Guidelines for the Care and Use of Laboratory Animals of China. The protocol was approved by the Ethics Committee of the First Hospital of Lanzhou University (Approval No. LDYYLL-2022-506).

### H&E staining and IHC

The samples were embedded in paraffin and cut into 4-μm-thick sections. The sections were dewaxed in xylene, hydrated in a graded alcohol series, and washed with phosphate-buffered saline for H&E staining. For IHC analysis, the slides were heated with 10 mM sodium citrate (pH 6.5) in a pressure cooker for 15 min. The nonspecific antigens were blocked with catalase enzyme body for 10 min, and 10% normal goat serum was added for 10 min. Then, the slices were incubated overnight at 4 °C with primary antibodies against CK7 (MAB-0828, 1:300, China), CK19 (MAB-0829, 1:300, China), Ki67 (MAB-0672, 1:1000, China), and p53 (MAB-0674, 1:500, China). Excess primary antibodies were washed off with PBS, and then, the slices were incubated with secondary antibodies for 30 min for DAB color development. Finally, counterstaining was performed with hematoxylin. The slides were observed using an Olympus DP26 light microscope.

### RNA sequencing analysis

To explore the transcriptome changes in CBC3T-1 cells, normal bile duct cells HIBEpiC were used as a control. Total RNA was extracted from frozen cell pellets using an RNeasy Micro Kit (Qiagen, CA) according to the manufacturer’s protocol. Then, RNA sequencing (RNA‐seq) was performed using the BGISEQ‐500 platform at BGI Genomics (Wuhan, China). The library preparation followed BGI’s standard procedure.

### Differential gene expression analysis

Differentially expressed genes (DEGs) were filtered and analyzed according to the following criteria: condition setting (|fold change|> = 2, *Q* value < 0.05). We used the BGI online platform (https://biosys.bgi.com/) to analyze the Gene Ontology (GO) and Kyoto Encyclopedia of Genes and Genomes (KEGG) pathways of the DEGs.

### Whole‑exome sequencing (WES) of CBC3T-1 cells

Sequencing and data analysis were performed at BGI (Wuhan, China). Briefly, CBC3T-1 cells were aligned with samples of adjacent normal tissue from this patient’s resected tumor tissue and genomic DNA extraction was performed. Library construction and whole‑exome capture of genomic DNA were performed using SureSelect Human All Exon V6 (Agilent), and the captured DNA library was sequenced on the DNBSEQ platform. Clean reads were aligned to the reference human genome (build hg19) using the Burrows‒Wheeler Aligner. To identify somatic mutations, we compared CBC3T-1 cells with adjacent normal tissue, filtering out germline mutations in normal tissue and retaining only those somatic mutations found in tumor cells during the analysis.

### Driver gene analysis

We compared somatic mutations with known driver genes in databases and the literature and screened out known driver genes in tumor samples. The reference data sources were Integrative OncoGenomics (IntOGen), Cancer Gene Census (CGC), three highly cited articles [14–16], and pan-cancer data [17].

### Statistical analysis

Statistical significance was calculated by unpaired two-tailed Student’s *t* test using GraphPad Prism 8 (GraphPad Software, Inc.). The data are presented as the means and standard deviations (SD) from at least three replicate analyses (bars). *P* < 0.05 was considered to indicate a statistically significant difference.

## Results

### Establishment and authentication of the CBC3T-1 cell line

We established a cell line from the primary tumor tissue of a 71-year-old male patient with hCCA and designated it CBC3T-1. The clinical and pathological features of the patient are shown in Fig. 1f. To date, CBC3T-1 cells have been maintained in single-molecule culture for over 60 generations. We performed STR analysis of 21 loci to determine the identity of the cell line and to exclude cross-contamination. The genomic identity of the CBC3T-1 cell line was also confirmed by comparison with the genetic profile of the primary tumor (Table 1). These results suggest that CBC3T-1 is a novel hCCA cell line. The cell line was maintained at the China Center for Type Culture Collection (CCTCC No: C2022169).

**Table 1:** STR profile of tumor tissue and cell line

Marker	CBC3T-1	Tumor tissue
D19S433	13, 15.2	13, 15.2
D5S818	9, 10	9, 10
D21S11	30, 31.2	30, 31.2
D18S51	13, 22	13, 22
D6S1043	11	11
AMEL	X, Y	X, Y
D3S1358	16	16
D13S317	8	8
D7S820	12	12
D16S539	9, 11	9, 11
CSF1PO	10	10
Penta D	11, 13	11, 13
D2S441	12	12
vWA	14, 17	14, 17
D8S1179	14, 15	14, 15
TPOX	8, 11	8, 11
Penta E	11	11
TH01	7	7
D12S391	22	22
D2S1338	19, 25	19, 25
FGA	23	23

### Characteristics of CBC3T-1 cells

**Fig. 2:**
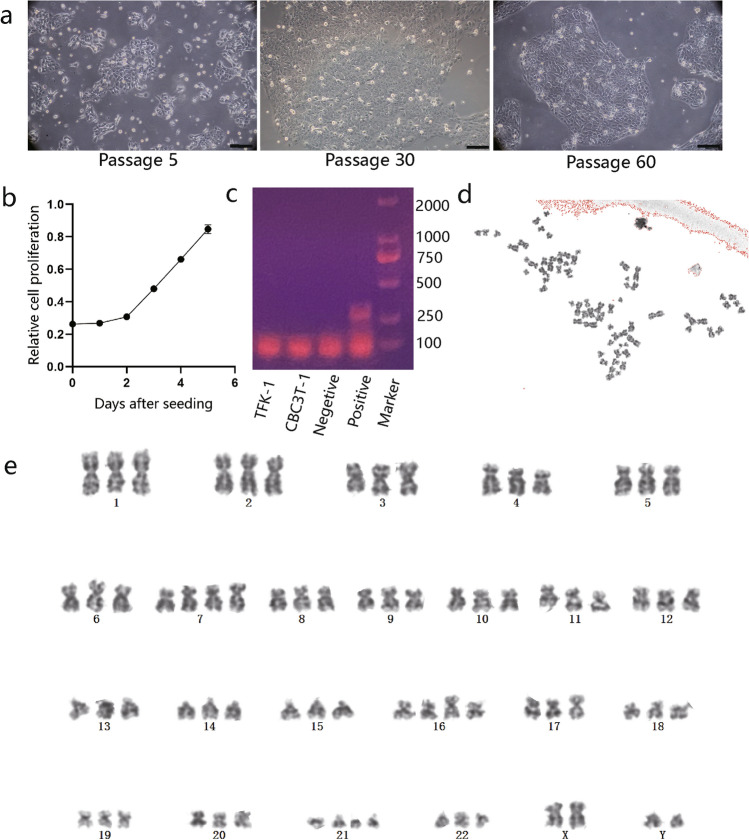
Characterization of CBC3T-1. **a** Brightfield morphology of CBC3T-1 cells in the 5th, 40th, and 60th passages (scale bars, 100 µm). **b** Cumulative growth curve of CBC3T-1 cells. **c** Mycoplasma test results. **d**, **e** Karyotype analysis of CBC3T-1 cells

CBC3T-1 cells were irregularly polygonal, with one or more oval nuclei and visible nucleoli (Fig. 2a). When grown at high density, the cells accumulated and showed loss of contact inhibition, indicating cellular malignancy. The doubling time of CBC3T-1 cells was 52 h (Fig. 2b). Meanwhile, we performed live cell imaging of CBC3T-1 cells to dynamically observe cell proliferation (Fig. S1a, b). CBC3T-1 cells were not contaminated with mycoplasma (Fig. 2c). Karyotype analysis of a representative single cell from CBC3T-1 shows a hyperploid cell line with a karyotype abnormality and a chromosome number between 69 and 73 (Fig. 2d, e).

### Characterization of CBC3T-1 cell behavior

**Fig. 3:**
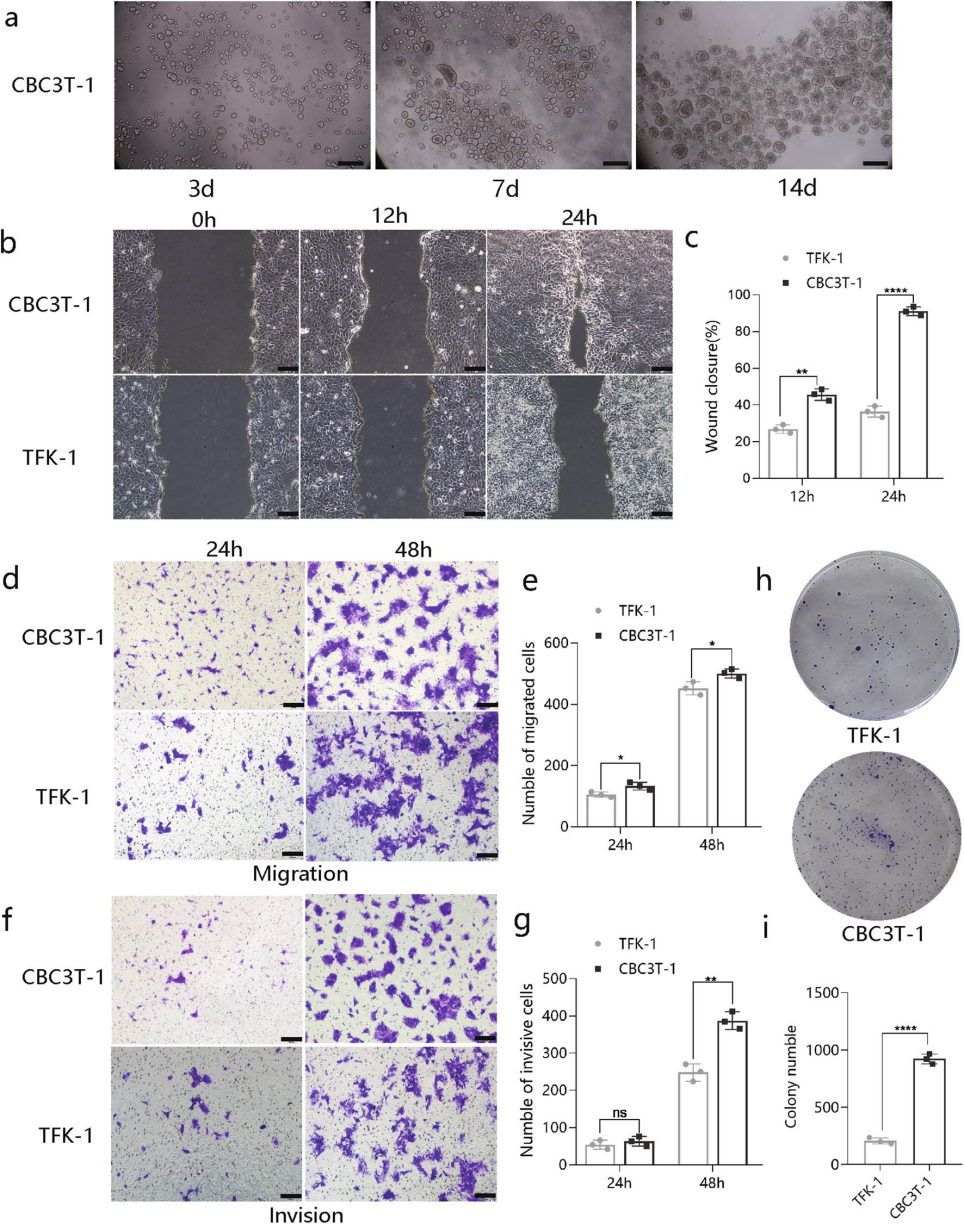
Characterization of CBC3T-1 cell behavior. **a** Representative images of CBC3T-1 spheres were obtained on days 1, 7, and 14. **b**, **c** Wound-healing assays at 12 and 24 h after scratching (*N* = 3). **d**–**g** Transwell assays were used to examine the cell migration and invasion capacity of CBC3T-1 and TFK-1 cells (*N* = 3). **h, i** Representative images showing colony formation (*N* = 3). Scale bars, 100 μm

We also assessed the ability of established CBC3T-1 cells to form spheres, with cells forming spheres in a low-adherent plate. The spheroid formation assay can be used to preliminarily judge the tumorigenicity of cells in vitro (Fig. 3a). To further characterize the tumorigenic properties of the CBC3T-1 cell line, TFK-1 cells were used as controls for further analysis. Wound-healing assay results showed higher levels of wound repair by CBC3T-1 cells at 12 h or 24 h (Fig. 3b, c). In addition, CBC3T-1 cells had a stronger migratory and invasive capacity than TFK-1 cells (Fig. 3d–g). Finally, we performed colony formation assays and found that CBC3T-1 cells exhibited enhanced clonogenic capacity (Fig. 3h, i).

### Sensitivity to anticancer agents

**Fig. 4:**
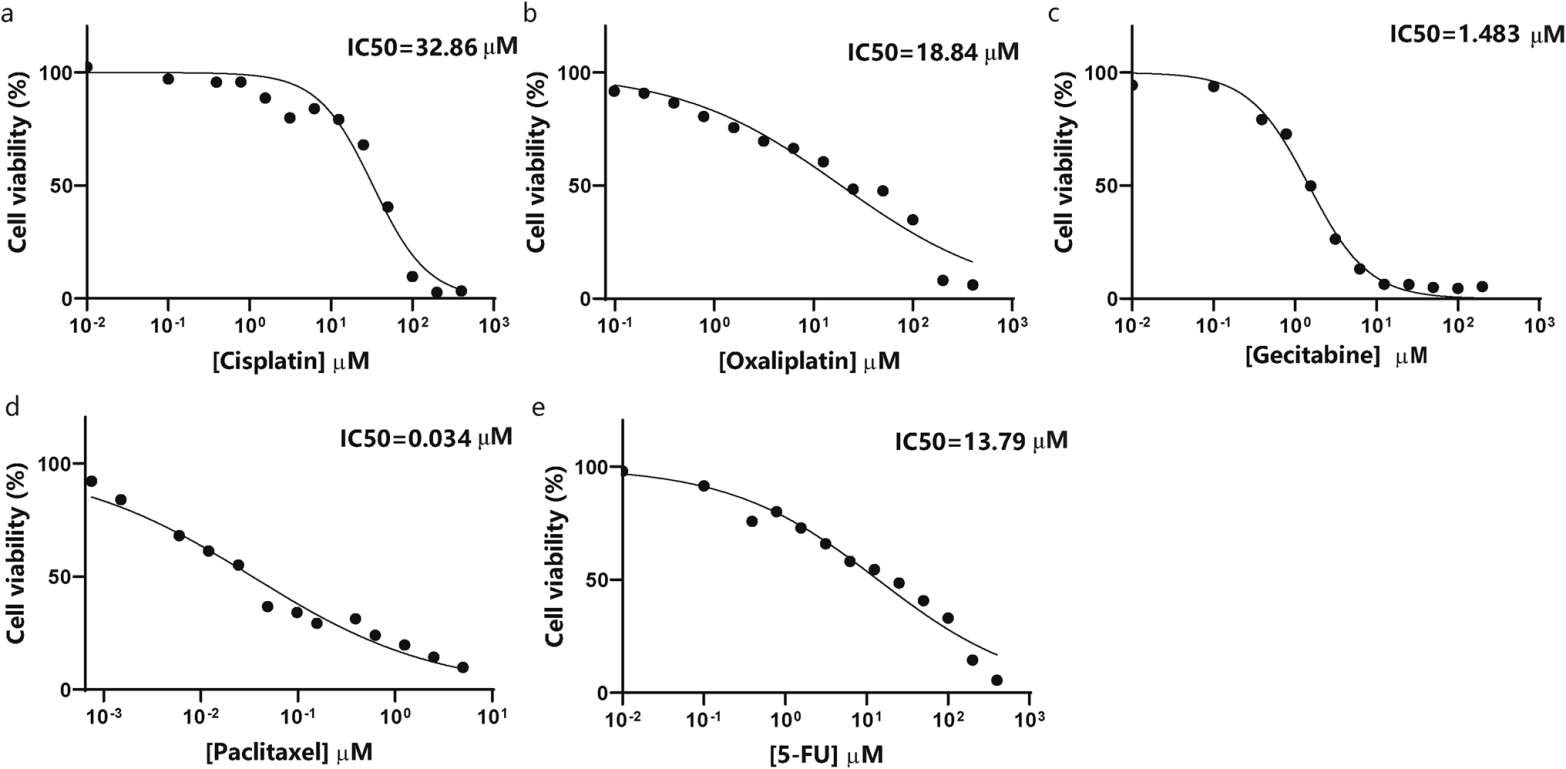
Cell viability of CBC3T-1 cells after 72 h of exposure to anticancer drugs. **a–e** Sensitivity of CBC3T-1 cells to cisplatin, oxaliplatin, gemcitabine, paclitaxel, and 5-FU

CCA has a poor response to currently available chemotherapeutic agents and a poor prognosis, mainly due to a lack of effective drug screening. CBC3T-1 cells were assessed for sensitivity to first-line anticancer drugs for the treatment of CCA. The IC50 values of cisplatin, oxaliplatin, gemcitabine, paclitaxel, and 5-FU were 32.86 µM, 18.84 µM, 1.483 µM, 0.034 µM, and 13.79 µM, respectively (Fig. 4a–e). Our results demonstrated that among these five anticancer drugs, paclitaxel had the best sensitivity, followed by gemcitabine.

### Tumorigenicity in vivo

**Fig. 5:**
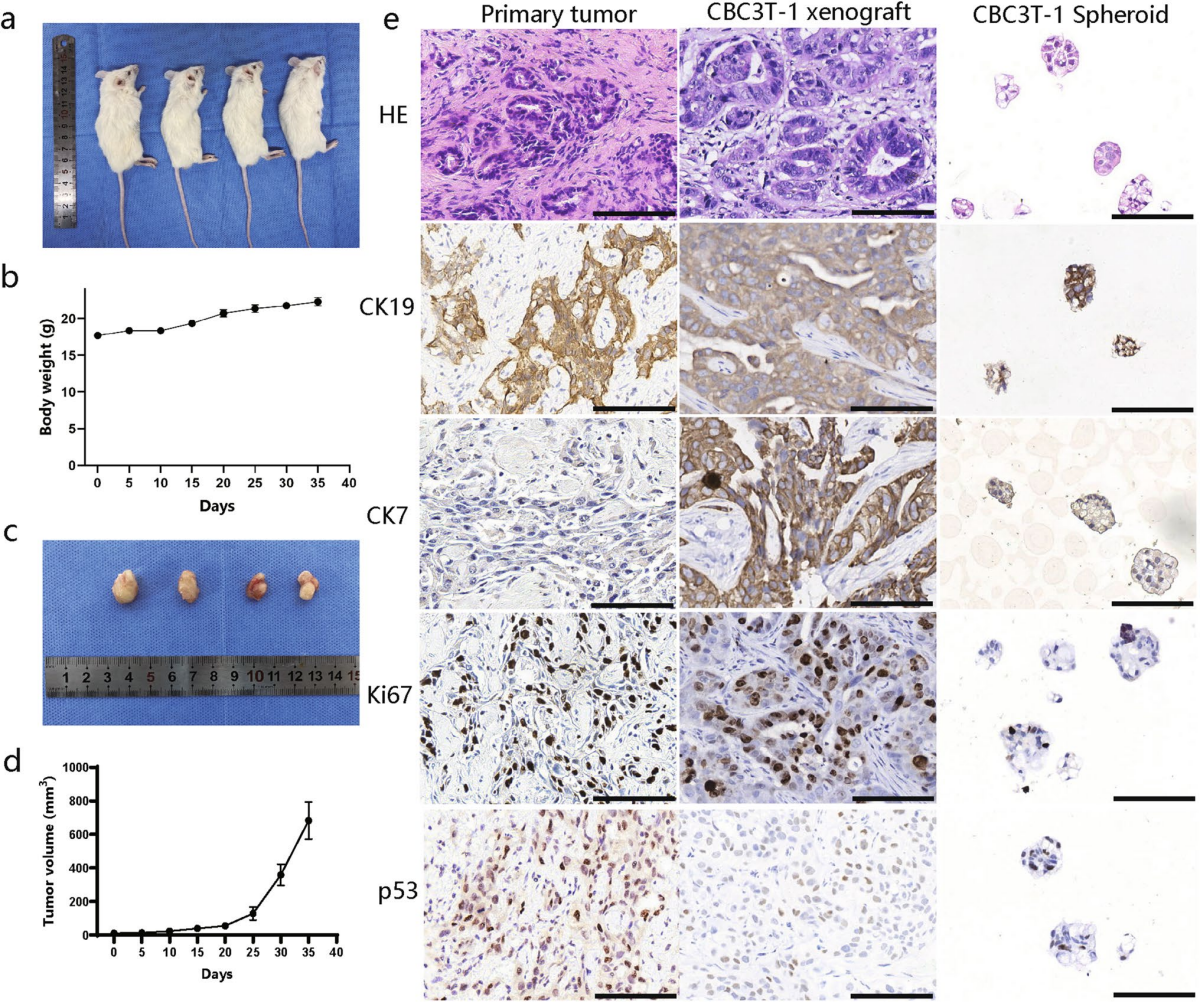
Tumorigenicity of the CBC3T-1 cell line in vivo. **a** Tumor formation in NOD/SCID mice. **b** Body weight gain curves in xenograft mice. **c** Tumor size in 4 xenograft mice. **d** Tumor growth curves of CBC3T-1 cells. **e** H&E staining and immunohistochemical (CK19, CK7, Ki67, and p53) results of primary tumors, CBC3T-1 xenografts, and CBC3T-1 spheroids. Scale bars, 100 μm

To determine whether CBC3T-1 cells are tumorigenic in vivo, we performed xenograft transplantation. All mice developed tumors after subcutaneous implantation of CBC3T-1 cells, and the tumors grew rapidly, with no significant change in body weight (Fig. 5a–d). Xenografts, the patient's primary tumor tissue and CBC3T-1 spheroids, were positive for the expression of CK7, CK19, Ki67, and p53 (Fig. 5e), demonstrating a biliary origin with p53 mutations. Ki67 expression indicated that the primary tumor tissue, xenografts and in vitro cultured CBC3T-1 spheroids were in a malignant proliferative state. These results indicate that the in vitro cultured cell lines faithfully recapitulate and maintain the expression status of the primary tumor and demonstrate the substantial tumorigenicity of CBC3T-1 cells.

### Analysis of important KEGG pathways and GO terms

**Fig. 6:**
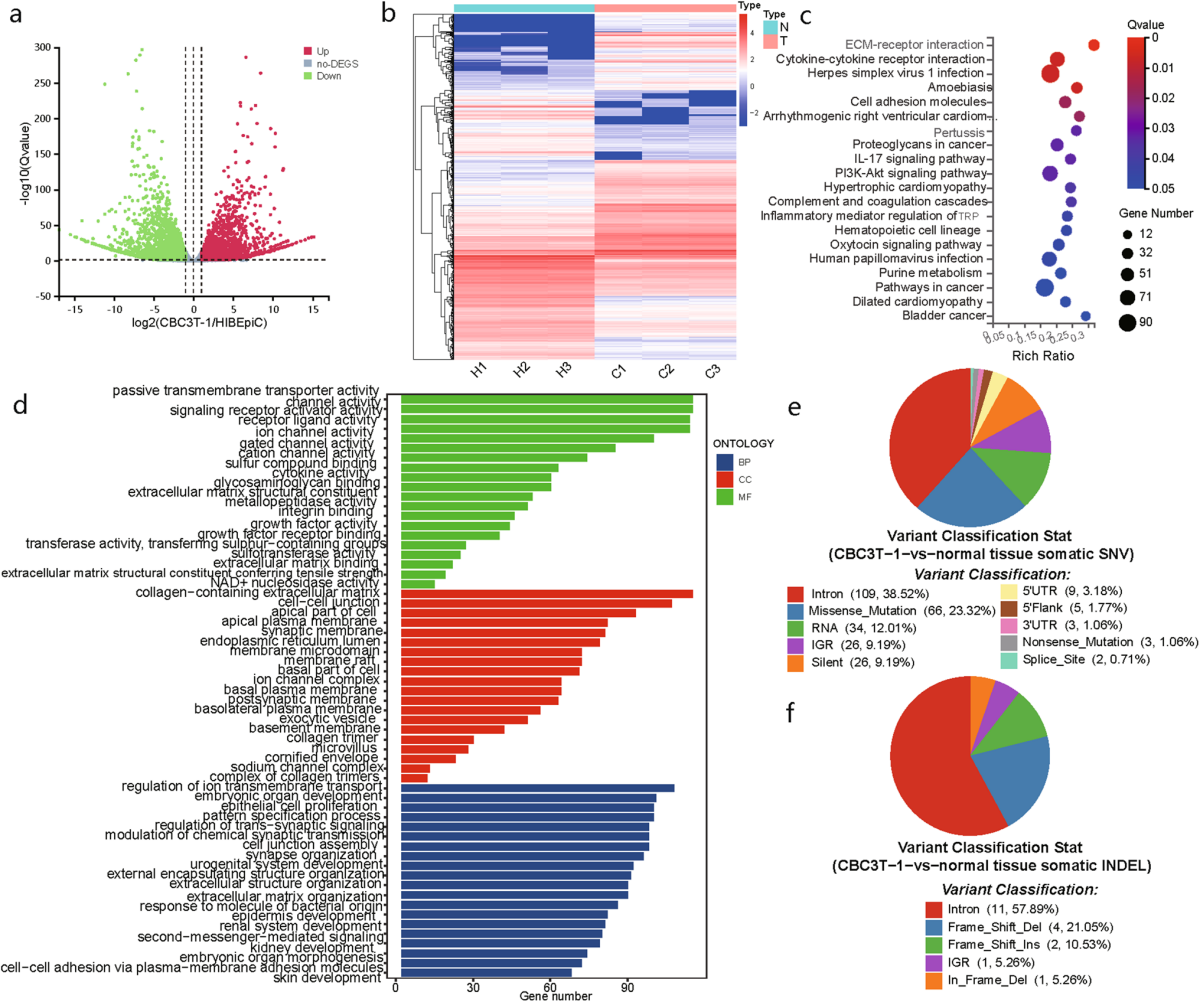
RNA-seq and WES of CBC3T-1 cells. **a** Differentially expressed genes between CBC3T-1 and HIBEpiC (|log2FC|> = 1; *Q* value < 0.05). **b** Heatmaps of up- and downregulated DEGs (|log2FC|> = 2; *Q* value < 0.01). **c** KEGG analysis reveals the top 20 genomic pathways for DEGs.** d** GO analysis revealed significant enrichment of DEGs in the top 20 terms of the three main functions.** e** Somatic SNV annotation result distribution map. **f** Somatic InDel annotation result distribution map

To study the evolution of the CBC3T-1 cell line, we performed RNA‐seq analysis. Of these DEGs, 2705 were upregulated and 3151 were downregulated (|fold change|> 2 and *p* < 0.05) (Fig. 6a). To further analyze the DEGs, we adjusted the screening conditions to a |fold change|> 4 and *p* < 0.01, and of the 2732 candidate genes detected, 1352 were upregulated and 1376 were downregulated (Fig. 6b).

KEGG pathway enrichment analysis showed that many cancer-related pathways were enriched in the CBC3T-1 cell line, including ECM–receptor interaction, cytokine‒cytokine receptor interaction, and the PI3K–Akt signaling pathway (Fig. 6c). The PI3K/AKT signaling pathway plays an important role in CCA carcinogenesis [18].

GO analysis was classified into three different categories: biological process (BP), cellular component (CC), and molecular function (MF) (Fig. 6d). Based on the functional enrichment results, the most enriched BP terms included regulation of ion transmembrane transport, embryonic organ development, and epithelial cell proliferation. CC terms included collagen-containing extracellular matrix and cell‒cell junction. Passive transmembrane transporter activity, channel activity, and signaling receptor activator activity were the most enriched MF terms.

### Whole‑exome sequencing analysis

To characterize the genomes of the established CBC3T-1 cell line, we performed WES of CBC3T-1. A total of 283 somatic single-nucleotide variants (SNVs) and 19 somatic InDels were identified through WES (Fig. 6e, f). We detected germline mutations in the normal tissues of patients based on GATK and then detected the mutated gene with CGC database comparison, screening for possible cancer susceptibility genes (Table 2). The main tumor susceptibility genes in CBC3T-1 cells included BARD1, KDR, FAT1, HNF1A, BRCA2, FANCA, and BRCA1. A study has reported that BRCA2 gene mutations are clinically relevant in patients with eCCA [19, 20]. In addition, the BRCA mutation frequency varies with biliary tract cancer subtype, as eCCA has a higher prevalence of BRCA mutations (4.8%) than iCCA (3.1%) [21].

**Table 2:** Susceptibility gene screening results

Gene	Chrom	Ref	Alt	Type	AA change	CGC-cancers
BARD1	chr2	G	A	Missense	p.Pro24Ser/c.70C>T	Ovarian cancer; breast cancer; endometrioid cancer
KDR	chr4	T	A	Missense	p.Gln472His/c.1416A>T	Melanoma
KDR	chr4	C	T	Structural interaction	c.889G>A	Melanoma
FAT1	chr4	T	C	Missense	p.Asn1664Ser/c.4991A>G	Pancreatic
HNF1A	chr12	T	C	Missense	p.Leu551Ser/c.1652T>C	Hepatic adenoma; hepatocellular carcinoma
BRCA2	chr13	A	C	Missense	p.Asn289His/c.865A>C	Breast; ovarian; pancreatic; leukemia
FANCA	chr16	G	A	Missense	p.Ser1088Phe/c.3263C>T	AML; leukemia
BRCA1	chr17	T	C	Missense	p.Ser1634Gly/c.4900A>G	Breast; ovarian
BRCA1	chr17	T	C	Missense	p.Glu1038Gly/c.3113A>G	Breast; ovarian

*Chrom* chromosome, *POS* position on chromosome, *REF* reference base, *ALT* alternative base, *AA* change: information on amino acid changes, *CGC-Cancers* tumor name annotated in the CGC database

The tumor genome contains numerous somatic mutations. However, only a few of them drive tumorigenesis by affecting genes. We compared somatic mutations with known driver genes in databases and the literature and screened out known driver genes in CBC3T-1 (Table 3). Susceptibility factors for hCCA and dCCA revolve around chronic inflammation within the larger bile ducts, mainly including primary sclerosing cholangitis (PSC) and liver fluke infection [22, 23]. Of these genes, TP53 and KRAS are the most commonly reported genetic alterations, with KRAS mutations being more common in hCCA than TP53 mutations [24–26]. These data provide a comprehensive description of the CBC3T-1 cell line as a distinct molecular entity.

**Table 3:** Results of screening for known driver genes

Gene	Chrom	Ref	Alt	Classification	AA change
PREX2	chr8	–	T	Frame_Shift_Ins	p.S565fs
KDM6A	chrX	G	–	Frame_Shift_Del	p.A694fs
PTPRC	chr1	C	T	Nonsense_Mutation	p.Q1036*
LTBP1	chr2	C	T	Intron	–
EBF1	chr5	G	A	Intron	–
DST	chr6	A	C	Intron	–
EEF1A1	chr6	C	G	Missense_Mutation	p.E268Q
CARD11	chr7	G	A	Silent	p.I398I
AMPH	chr7	G	A	5'UTR	–
TRIM24	chr7	C	G	Intron	–
PDCD1LG2	chr9	G	A	Missense_Mutation	p.R181H
MAPK8IP1	chr11	G	A	Intron	–
ZBTB16	chr11	G	A	Missense_Mutation	p.M348I
KRAS	chr12	C	A	Missense_Mutation	p.G12V
MGA	chr15	C	G	Silent	p.L2623L
TP53	chr17	C	T	Missense_Mutation	p.E285K
ZNF521	chr18	C	T	Silent	p.A182A
TSHZ3	chr19	C	T	Silent	p.S837S
DMD	chrX	G	A	Intron	–

*Chrom* chromosome, *REF* reference base, *ALT* alternative base, *AA change* information on amino acid changes

## Discussion

Patient-derived cell lines facilitate basic research to elucidate the mechanisms of disease development and to screen new antitumor drugs. Given the aggressive nature of CCA, the establishment of new well-defined and well-characterized CCA cell lines is crucial. Although eCCA cell lines, namely, ZJU-0826 and QBC939, have been previously reported, the number established in the Chinese population is too small to cover the full range of features of eCCA [11, 27, 28].

In this study, we established and assessed the characteristics of an hCCA cell line, CBC3T-1, derived from a high-incidence region in China. The STR profile confirmed that CBC3T-1, a newly established cell line of human origin. Mycoplasma is a bacterium capable of infecting human cancer cell lines. Mycoplasma contamination alters a myriad of characteristics of infected cell lines from metabolism to gene expression [29, 30]. Therefore, we tested CBC3T-1 cells uninfected with mycoplasma. In addition, CBC3T-1 cells proliferated rapidly, with a doubling time of 52 h and a high stemness capacity to form spheres. In an in vivo mouse model, the CBC3T-1 cell line was capable of subcutaneous tumor formation and maintained the characteristics of the primary tumor.

Patient-derived cell lines are a valuable research tools. They are not fully representative of clinical tumor models. Therefore, we need extensive multiomics studies to determine the extent to which patient-derived cell lines can be used. We performed RNA sequencing analysis on CBC3T-1 and HIBEpiC. Our data showed 1352 upregulated genes and 1376 downregulated genes. These genes were mainly enriched mainly in pathways associated with tumorigenesis and development. The PI3K/AKT/mTOR pathway has been reported to be a central regulator of cell metabolism, growth, and survival, and is involved in the development and progression of CCA [31, 32]. Numerous studies have been reported on PI3K/AKT inhibitors, but the objective response rate for bile duct cancer is low [31].

Upon performing WES, we screened for somatic mutations. Among these cancer susceptibility genes, mutations in the BRCA2 gene occurred at a low frequency in patients with CCA, mainly in the intrahepatic locus. BRCA2 mutation carriers have an increased risk of pancreatic, gallbladder, and bile duct cancers [33]. In addition, the TP53 and KRAS genes are common drivers of eCCA and play an important role in the CBC3T-1 cell line. TP53 and KRAS mutations have been found to have a negative impact on the prognosis of biliary tract carcinoma [34]. However, in one study, there were differences in the genomic profiles of Chinese and Western eCCA patients, with almost twice the prevalence of KRAS in the MSKCC cohort compared to the Chinese cohort [35]. This also suggests the need to establish cell lines from different populations.

This study has some limitations and should be further refined in the future. Long-term in vitro cultures of CCA cells may be subject to certain genetic changes that may affect the reproducibility of the data from subsequent studies. As the cells continue to grow in passages, we need to study the changes in their properties from the original cells. In addition, the currently established eCCA cell lines do not further differentiate between those originating from hCCA or dCCA, and thus failed to include more hCCA cell lines for comparative analysis. CCA originating from different sites are highly heterogeneous, and therefore, the origin should be clarified according to the anatomical site in future studies.

In conclusion, we generated and characterized a new hCCA cell line named CBC3T-1, which was stable for at least 17 months. CBC3T-1 has important characteristics of tumor cells from CCA patients and can be used to study the molecular mechanisms of CCA, discover new antitumor compounds, and show the clinical utility of targeted drugs.

### Supplementary Information

Below is the link to the electronic supplementary material.

Supplementary file1 Supplementary Fig. S1 Live cell imaging analysis of CBC3T-1 cells. a Representative images of CBC3T-1 cell proliferation at different time points. b Eleven representative images of CBC3T-1 cell proliferation (0, 12, 24, 36, 48, 60, 72, 84, 96, 108, and 120 h). Scale bars, 200 μm (TIF 21736 KB)

## Data Availability

The datasets used and/or analyzed in this study are available from the corresponding authors upon reasonable request.
