# Supplementary figures and images for: Establishment and characterization of a novel hilar cholangiocarcinoma cell line, CBC3T-1
Source: Hum Cell. 2023 Nov 15;37(1):364–75. doi: 10.1007/s13577-023-01003-4 (PMC10764469; doi:10.1007/s13577-023-01003-4)

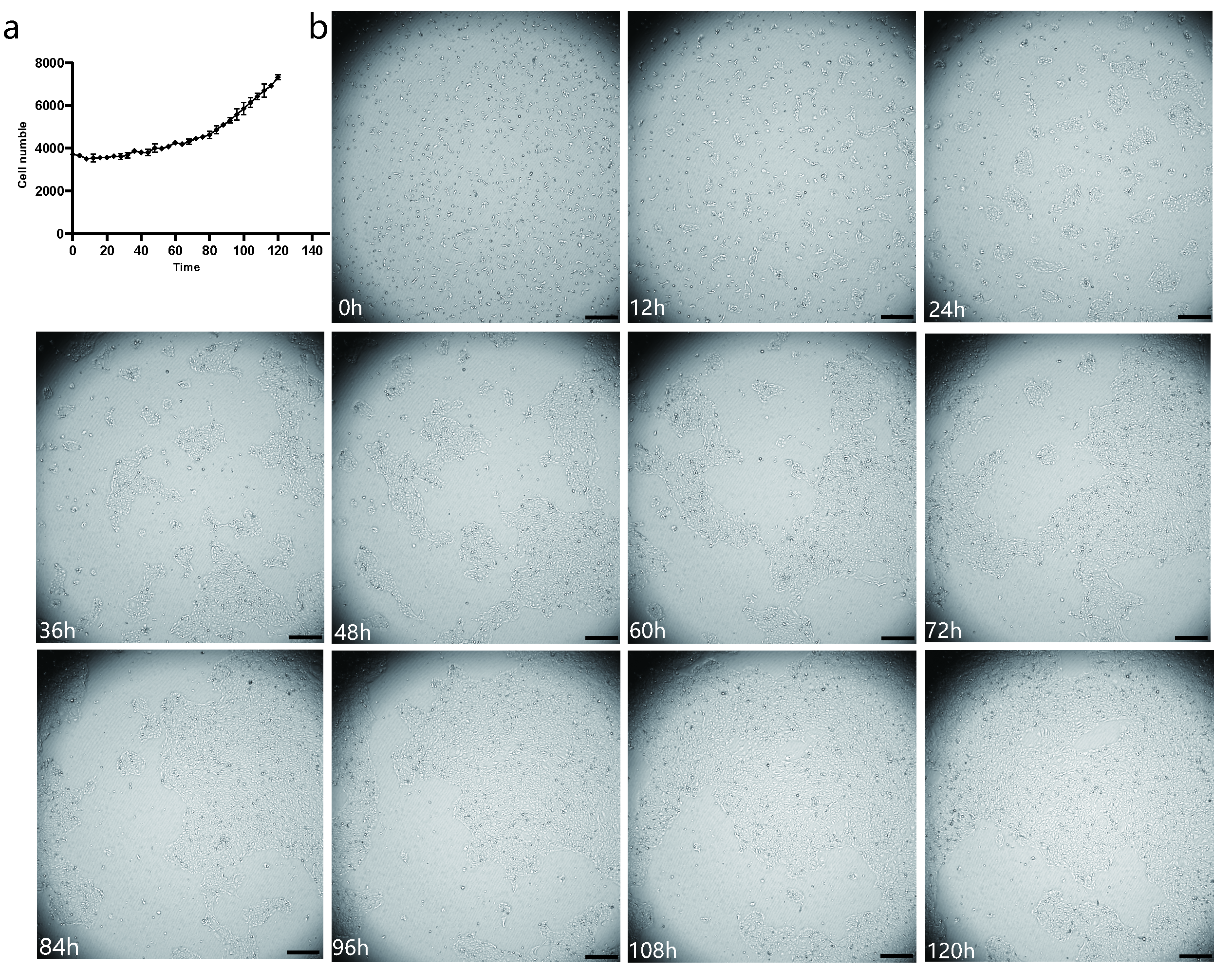

Supplement: Supplementary file 1 — Supplementary file1 Supplementary Fig. S1 Live cell imaging analysis of CBC3T-1 cells. a Representative images of CBC3T-1 cell proliferation at different time points. b Eleven representative images of CBC3T-1 cell proliferation (0, 12, 24, 36, 48, 60, 72, 84, 96, 108, and 120 h). Scale bars, 200 μm (TIF 21736 KB) [file 13577_2023_1003_MOESM1_ESM.tif]
